# Positive expression of NANOG, mutant p53, and CD44 is directly associated with clinicopathological features and poor prognosis of oral squamous cell carcinoma

**DOI:** 10.1186/s12903-015-0120-9

**Published:** 2015-12-01

**Authors:** Hye-Jin Lee, Young-Hoon Kang, Jong-Sil Lee, June-Ho Byun, Uk-Kyu Kim, Si-Jung Jang, Gyu-Jin Rho, Bong-Wook Park

**Affiliations:** Department of Oral and Maxillofacial Surgery, School of Medicine and Institute of Health Science, Gyeongsang National University, Jinju, 660-702 Republic of Korea; Department of Pathology, School of Medicine, Gyeongsang National University, Jinju, Republic of Korea; Department of Oral and Maxillofacial Surgery, School of Dentistry, Pusan National University, Busan, Republic of Korea; OBS/Theriogenology and Biotechnology, College of Veterinary Medicine, Gyeongsang National University, Jinju, Republic of Korea

**Keywords:** Oral squamous cell carcinoma, Tumor markers, Immunohistochemistry, NANOG, Mutant p53

## Abstract

**Background:**

In order to predict long-term prognosis and define individual treatment modalities for patients with oral squamous cell carcinoma (OSCC), more reliable tumor biomarkers are needed during the pretreatment workup period. The present study aimed to identify more reliable immunohistochemical tumor prognostic markers in the pretreatment biopsy specimens of patients with OSCC.

**Methods:**

We selected 57 patients who were diagnosed with primary OSCC through histopathological analysis. Pretreatment biopsy specimens were immunohistochemically analyzed for the transcription factor NANOG, cancer stem cell marker CD44, and mutant tumor protein 53 (mutant p53). The immunostaining patterns were assessed for their association with the clinicopathological features of OSCC and overall survival rates.

**Results:**

Late tumor stage, positive neck node metastasis, and high-grade differentiation were associated with significantly poorer survival rates. Enhanced expression of NANOG and mutant p53 positivity were significantly associated with clinically late-stage tumors, positive neck node metastasis, histologically high-grade tumors, and poor overall survival rates. OSCCs with strong co-detection of NANOG and mutant p53 were linked to significantly lower survival rates than those with both weak NANOG expression and p53 negativity. Increased expression of CD44 had a limited correlation with unfavorable clinicopathological features.

**Conclusion:**

High expression of NANOG and positive expression of mutant p53 in the pretreatment biopsy specimens of patients with OSCC were associated with poor survival rates and unfavorable clinicopathological features. These results demonstrate that NANOG, mutant p53, and CD44 could be used as immunohistochemical markers in the pretreatment specimens of OSCC. In particular, analysis for co-expression of NANOG and mutant p53 should be made highly available as a tool for prognosis and selecting individual treatment modalities.

## Background

Oral squamous cell carcinoma (OSCC) is a common malignant tumor of the head and neck region; however, patients with OSCC have a 5-year survival rate of less than 50 %. The traditional TNM staging and histopathological grading systems do not enable physicians to accurately predict a tumor’s aggressiveness or select treatment modalities on an individual basis [1]. Therefore, research has been underway to investigate more effective and definitive tumor prognostic markers for patients with OSCC [2, 3]. As the cancer stem cell (CSC) hypothesis has become one of the predominant theories explaining the tumor-initiating capacity and heterogeneity of tumor cells, various stem cell markers have been studied to determine their correlation with clinicopathological tumor features and long-term prognosis [4–8].

The early transcription factors NANOG, OCT4, and SOX2, which play pivotal roles in the maintenance of pluripotency and self-renewal ability in both embryonic and adult stem cells, have also been reported as key regulation factors in the CSCs of head and neck squamous cell carcinoma (HNSCC) [7, 8]. High expression of NANOG and OCT4 has been positively correlated with histologically high-grade carcinomas and clinically poor prognosis [7–10]. CD44 was the first CSC marker described in a solid malignancy [5], and a high frequency of CD44 positive cells in HNSCC strongly correlates with recurrence and tumor aggressiveness [11]. Tumor protein 53 (p53) is considered a traditional tumor biomarker in squamous cell carcinoma (SCC); however, its usefulness as a prognostic indicator remains unclear [1]. Wild-type p53 protein is barely detectable in normal tissues because of its short half-life of approximately 20 min. [12] However, the tumor suppressor gene p53 is mutated in approximately 40–60 % of OSCC cases, and this mutant p53 plays an important role in tumor development and progression [12, 13]. Interestingly, mutant p53 protein is usually detectable via immunohistochemistry (IHC) [14, 15].

In order to predict long-term prognosis and define individual treatment modalities for patients with OSCC, more reliable tumor biomarkers (including immunohistochemical prognostic markers) are needed during the pretreatment workup period. The present study aimed to investigate the expression of various tumor markers via IHC, including stem cell and tumor-related biomarkers, to identify more reliable prognostic markers in OSCC biopsy specimens collected prior to cancer treatment. In the present study, we observed a positive correlation between the clinicopathological features of OSCCs and the immunohistochemical expression patterns of NANOG, human mutant p53, and CD44. In addition, the immunostaining intensities of these marker proteins (NANOG and mutant p53) were positively correlated with the overall survival rates of patients with OSCC.

## Methods

### Patients

Between 2004 and 2014, a total 92 patients were diagnosed with OSCC in the Department of Oral and Maxillofacial Surgery of Gyeongsang National University Hospital (GNUH) based on the histopathological analysis of biopsy specimens collected prior to cancer treatment. Among them, 57 patients with primary OSCC (excluding patients with metastatic and recurrent cancers; 36 men and 21 women) were selected for the present study. The inclusion criteria were patients who agreed to participate in the study, completed at least 3 months of follow-up after undergoing pretreatment biopsy, and had sufficient tissue volume in the pretreatment biopsy specimen to perform immunohistochemical staining. The medical records of the selected patients were retrospectively analyzed. Informed consent for the use of their tissue specimens was obtained from all patients, and this study was approved by the Ethic Committee for Clinical Research at GNUH (GNUH IRB-2012-09-004-002). IHC was performed on the pretreatment biopsy specimens of the 57 patients, which were obtained from the periphery of the tumor margin with a minimum length of 5 mm and minimum width and depth 2 mm, to analyze the relationship between protein expression patterns and clinicopathological tumor features. The duration of follow-up for the 57 patients ranged from 3 to 127 months, with a mean of 35.9 months. The patients were clinically evaluated according to the TNM classification system developed by the American Joint Committee on Cancer (7^th^ edition, 2010) for tumor stage and neck node metastasis, and overall survival rates were also determined. Biopsy specimens were histopathologically graded, and tumors were categorized as well, moderately, or poorly differentiated according to the WHO classification of tumors [16].

### Immunohistochemical analysis of biopsy specimens

Biopsy specimens were fixed in 10 % neutral buffered formalin for 24 h, embedded in a paraffin block, sliced into 4-μm sections and mounted on SuperfrostPlus microscope slides (Fisher Scientific, Rochester, NY, USA). The sections were maintained at room temperature for 12 h. After hydration, IHC for NANOG, CD44, and mutant p53 was performed using an automated immunostainer (BenchMark XT, Ventana Medical System Inc., Tucson, AZ, USA), and visualization was conducted using the Ultraview DAB kit (Ventana Medical System Inc.) according to the manufacturer’s protocol. For immunostaining of the three marker proteins, we used rabbit monoclonal antibodies (Abcam™, Cambridge, UK). The dilution rate and lot numbers of the primary antibodies are summarized in Table 1. Briefly, sections were deparaffinized using EZ Prep solution; then CC1 standard (pH 8.4 buffer contained Tris/Borate/EDTA) was applied for antigen retrieval for 60 min at 100 °C. The slides were incubated at 37 °C for 4 min with a DAB inhibitor (3 % H_2_O_2_) to block endogenous peroxidase activity. Then, slides were incubated with the primary antibody at 37 °C for 32 min, followed by incubation with the secondary antibody (Universal HRP Multimer) for 8 min at 37 °C. Slides were treated with DAB+ H_2_O_2_ substrate for 8 min followed by hematoxylin II and bluing reagent at 37 °C for nuclear counterstaining. For negative controls, only incubation with the secondary antibody was performed, omitting any incubation with a primary antibody, in sections from the same specimens under the same conditions. In addition, human embryonic urinary bladder and skin carcinoma tissues were used as positive controls for NANOG, CD44, and mutant p53, respectively, according to the antibody manufacturer’s recommendations.

**Table 1:** Summary of clinicopathological features of OSCCs and IHC expression patterns of NANOG, CD44, and mutant p53

		NANOG			CD44			Mutant p53	
	Total No.	+++	++	+&–	++	+	–	+	–
No. of cases	57	21	18	18	28	21	8	34	23
Tumor sites									
Gingiva	27	13	8	6	10	11	6	13	14
Cheek	4	0	3	1	2	2	0	3	1
Palate	10	4	3	3	9	1	0	10	0
Tongue	9	0	2	7	2	5	2	4	5
FOM	7	4	2	1	5	2	0	4	3
Tumor stage									
I + II	24	6	4	14	10	9	5	11	13
III + IV	33	15	14	4	18	12	3	23	10
Neck node									
N0	34	9	10	15	13	15	6	16	18
N+	23	12	8	3	15	6	2	18	5
Histo grade									
Well	22	4	4	14	9	9	4	6	16
Mod + Poor	35	17	14	4	19	12	4	28	7
Antibody									
Dilution rate		1:250			1:100			1:200	
Source (Lot No.)		Abcam™ (ab109250)			Abcam™ (ab51037)			Abcam™ (ab32049)	

Abbreviations: *FOM* floor of mouth, *N0* negative neck node, *N+* positive neck node, *Well* well-differentiated OSCC, *Mod + Poor* moderately and poorly differentiated OSCC

Tissue slices were semi-quantitatively analyzed for antibody deposition in cellular components by two pathologists who were blinded to the study information. Based on a previously reported method with modification [10], positive immunostaining of NANOG and CD44 was scored by the combination of intensity (0, negative staining; 1, weak staining; 2, moderate staining; and 3, strong staining) and the percentage of positively stained tumor cells in high-power fields (0, negative; 1, <25 %; 2, 25–50 %; 3, 51–75 %; and 4, >75 %). The sum of the staining intensity and percentage of positive tumor cell scores was graded as follows: +++ (strong, 6–7); ++ (moderate, 4–5); + (weak, 2–3); and − (negative, 0–1). The expression pattern of mutant p53 was also scored using the combination of staining intensity and the percentage of positive tumor cells and simply graded as + for positive (sum of the score exceeded 3) and − for negative (2 or less; negative expression or weak expression in less than 25 % of the cells), in line with previous reports [10, 17, 18]. At least three different fields under high magnification (×400) per slide were analyzed for immunostaining intensity. A summary of the immunohistochemical staining results can be found in Table 1.

### Statistical analysis and overall survival analysis

The relationship among the expression intensities of NANOG, mutant p53, and CD44 in OSCC specimens was statistically evaluated using the chi-squared test. Similarly, the correlation between the protein expression patterns and clinicopathological features of OSCC, including tumor stage, neck node metastasis, and histological grade, was also analyzed using the chi-squared test. The overall survival analysis was conducted using the Kaplan–Meier method, and the data were compared using a log-rank test for the 57 OSCC specimens. First, overall survival analysis was performed in terms of the treatment modality as well as clinicopathological tumor features, including tumor stage, neck node metastasis, and histological grade (Fig. 5a–d). In the present study, patients were divided according to treatment modalities into three groups: surgery only (Surg), surgery combined with adjuvant radiotherapy (Surg + RT, including concurrent chemoradiotherapy), and radiotherapy only (RT). Most patients in the RT group underwent palliative radiotherapy because they refused to undergo surgery or their tumors were inoperable. Second, survival analysis was conducted in terms of the immunostaining intensities of NANOG, mutant p53, and CD44 in the pretreatment biopsy specimens (Fig. 5e–h). Univariate and multivariate survival analyses were performed using a Cox proportional hazards regression model. All analyses were performed using IBM SPSS Statistics software (SPSS Inc., Chicago, IL, USA). The cases were censored at the date of either the patient’s death or the last follow-up. Results were considered significant at *p* < 0.05, and these differences were denoted by an asterisk or different letters.

## Results

### Information of patients selected

A total of 57 patients with OSCC consisting of 36 men and 21 women were included in this study. Patient age ranged 17–90 years (mean, 65.4 ± 13.9 years). The site of OSCC in the patients included the gingiva (27 cases), palate (10 cases), tongue (9 cases), floor of the mouth (7 cases), and cheek (4 cases) (Table 1). Patients were divided according to treatment modality as follows: 15 patients in the Surg group, 22 patients in the Surg + RT group, and 20 patients in the RT group. The mean follow-up period in all patients was 35.9 months; meanwhile, 24 patients (19, 4, and 1 patient in the RT, Surg + RT, and Surg groups, respectively) died after an average of 18.4 months, and 33 patients survived over an average follow-up of 48.7 months. In all cases of surgery, resection tumor margins were set as at least 2 cm, and the resection field was confirmed to be tumor-free using frozen biopsy sections.

### Expression patterns of NANOG, mutant p53, and CD44 in OSCC pretreatment biopsy specimens

**Fig. 1:**
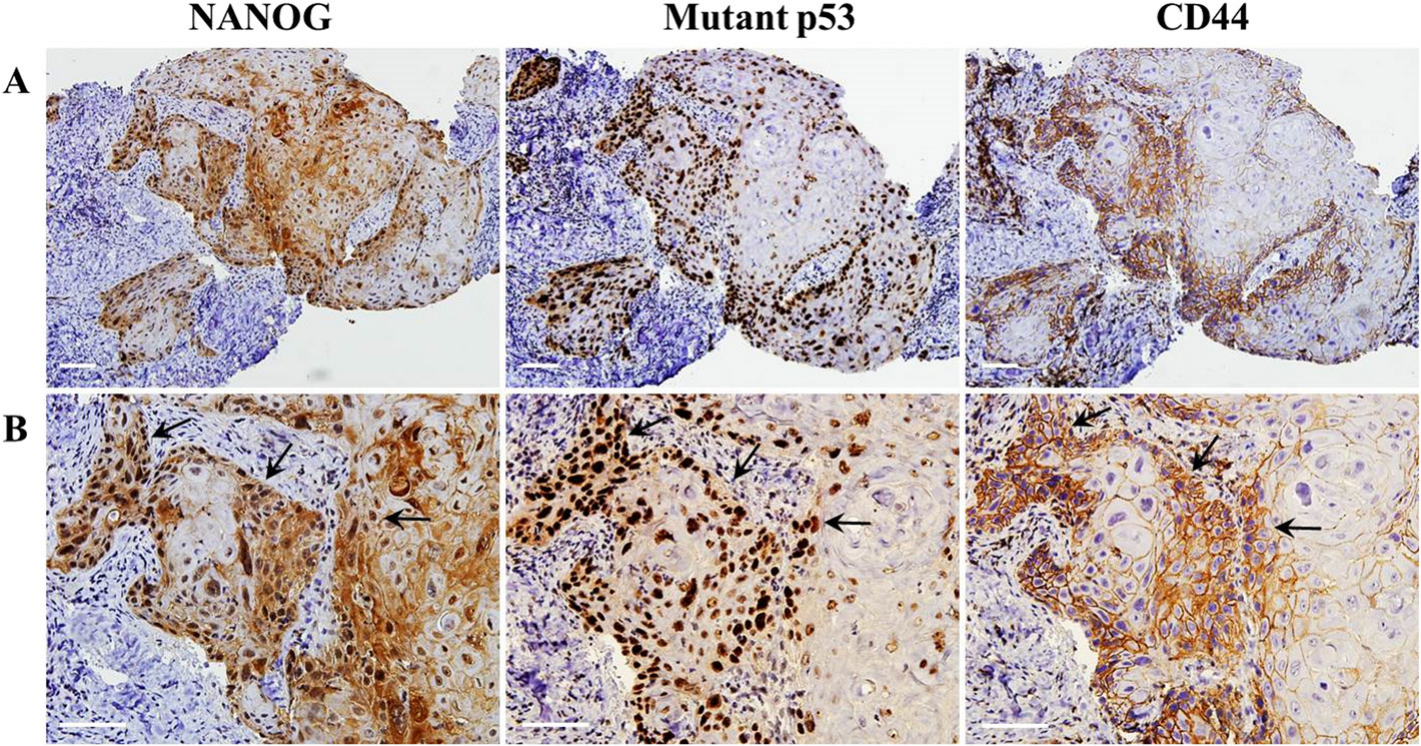
Immunohistochemical staining of NANOG, mutant p53, and CD44 in OSCCs. **a** Serial sections of moderately differentiated OSCC specimens show moderate expression of NANOG and CD44, and positive expression of mutant p53. **b** In higher magnification fields of serial sections, NANOG is detected mainly in the nuclei but is also expressed in the cytoplasm of some tumor cells. Mutant p53 is detected in the nuclei of tumor cells, while CD44 is localized in the membranes of cancer cells. The arrows indicate the co-expression sites of NANOG, mutant p53, and CD44 in OSCCs. Scale bar = 50 μm

**Fig. 2:**
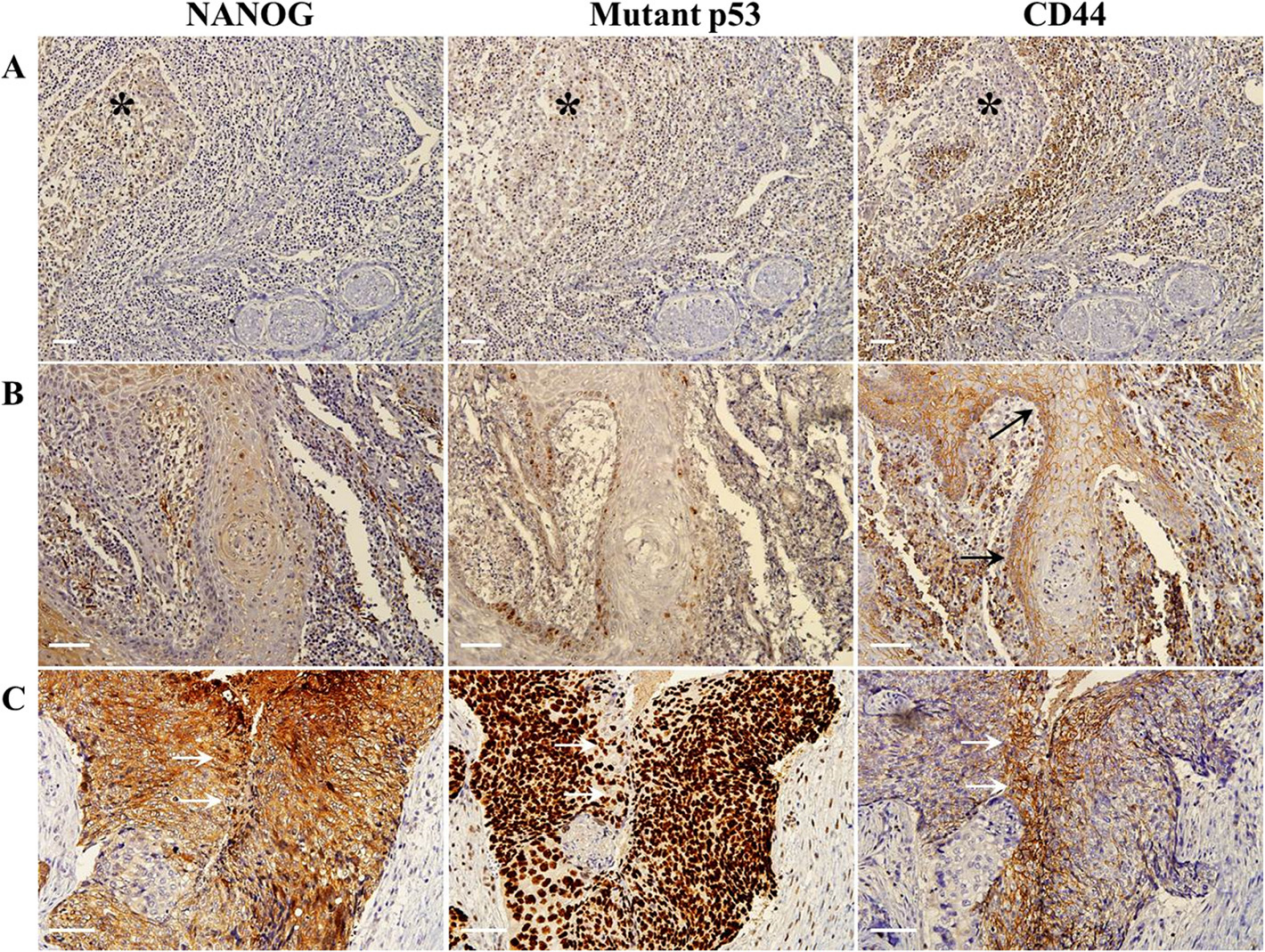
Immunostaining of well (**a **& **b**) and poorly (**c**) differentiated OSCCs. **a** In serial sections, the negative expression of NANOG, mutant p53, and CD44 is detected in one specimen of well-differentiated OSCC (* indicates cancer tissue). **b** In another case of well-differentiated OSCC, NANOG and mutant p53 are almost negative, seen in less than 25 % of positive cells, whereas CD44 is weakly detected in the cancer cell membrane (arrows). **c** In a poorly differentiated OSCC specimen, enhanced expression of NANOG, mutant p53, and CD44 is detected. The arrows indicate co-localization of the three marker proteins. Scale bar = 50 μm

NANOG was mainly detected in the nuclei of OSCC cells, but some NANOG expression was detected in the cytoplasm of cancer cells. Mutant p53 was localized in the nuclei of cancer cells, whereas CD44 was detected in the cell membrane of OSCC cells (Fig. 1). The number of positive cells and the expression intensities were usually greater in high-grade OSCCs (moderately or poorly differentiated) than in low-grade tumors (well-differentiated) (Figs. 1 and 2). Of the 57 OSCC specimens, the expression of NANOG was strong in 21 specimens, moderate in 18 specimens, and weak or negative in 18 specimens. Moderate, weak, and negative expression of CD44 was observed in 28, 21, and 8 OSCC specimens, respectively. In addition, 34 specimens were positive for mutant p53, whereas 23 specimens were negative (Table 1).

**Fig. 3:**
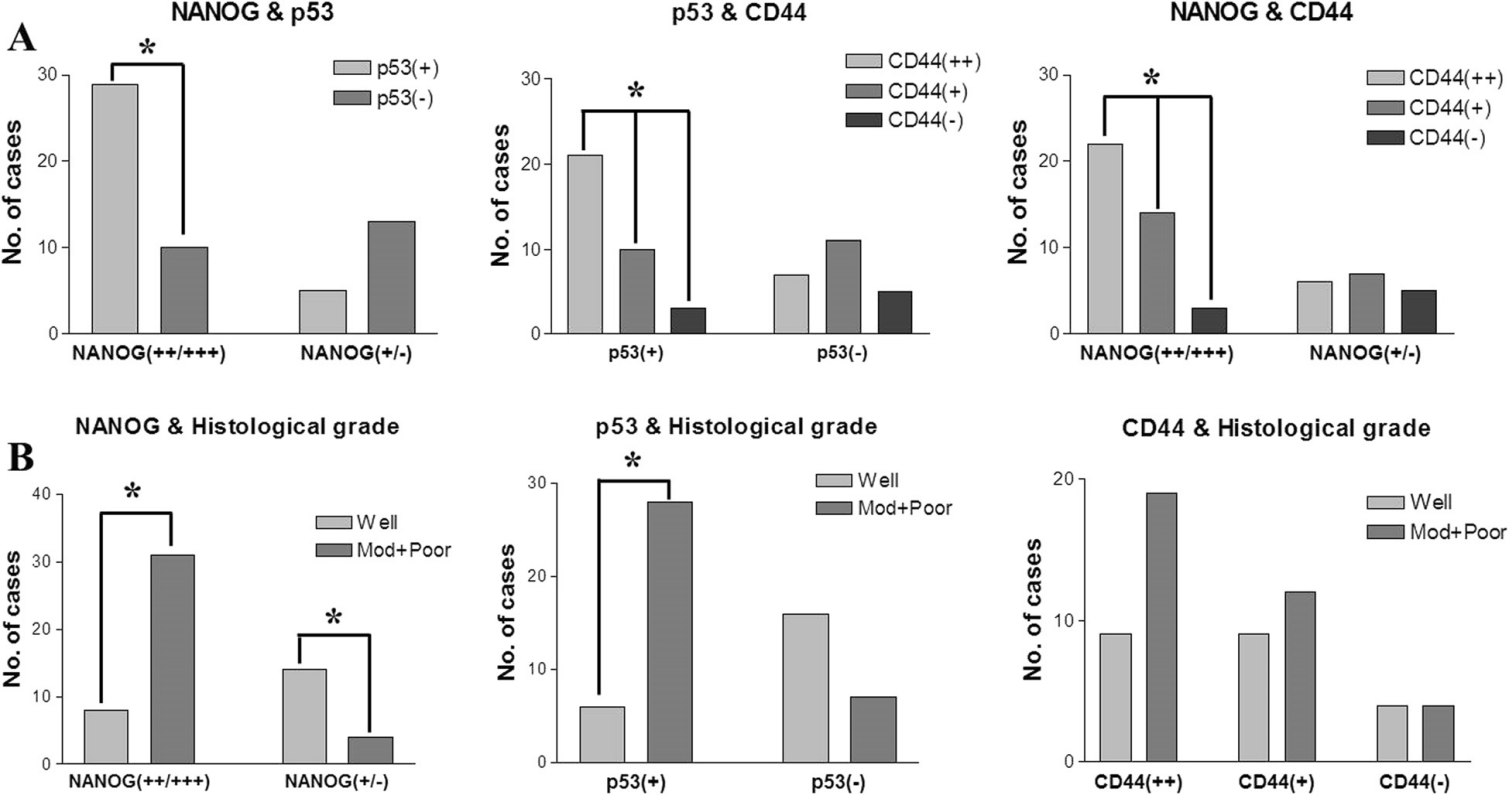
Correlation of the expression patterns of NANOG, mutant p53, and CD44 and the histological grade of OSCCs. **a** Tumors with enhanced expression of NANOG show a higher frequency of mutant p53 positivity (*p* = 0.002) and enhanced CD44 expression (*p* < 0.001). Similarly, OSCCs with positive expression of mutant p53 show higher expression of CD44 (*p* < 0.001). **b** Strong or moderate expression of NANOG is significantly related to histopathologically high-grade OSCCs (*p* < 0.001); however, weak or negative expression of NANOG correlates with well-differentiated carcinoma (*p* = 0.018). Moreover, positive mutant p53 expression is significantly associated with high-grade carcinoma (*p* < 0.001). Similarly, enhanced CD44 expression is associated with high-grade tumors, but no statistical significance was observed (*p* = 0.058). Data represent the number of cases with positive expression of each protein, and an asterisk (*) indicates significant differences (*p* < 0.05). [Abbreviations: NANOG(++/+++), strong or moderate expression of NANOG; NANOG(+/−), weak or negative expression of NANOG; p53(+), positive expression of mutant p53; p53(−), negative expression of mutant p53; CD44(++), more than moderate expression of CD44; CD44(+), weak expression of CD44; CD44(−), negative expression of CD44; Well, well-differentiated OSCCs; Mod + Poor, moderately and poorly differentiated OSCCs]

Direct relationships among the expression intensities of NANOG, mutant p53, and CD44 were observed (Fig. 3a). Tumors with strong expression of NANOG also frequently exhibited positive mutant p53 expression (*p* = 0.002) and enhanced CD44 expression (*p* < 0.001). Similarly, tumors with positive mutant p53 expression displayed enhanced expression of CD44 (*p* < 0.001) (Fig. 3a). In some cases, co-expression of NANOG, mutant p53, and CD44 was detected in serial sections of the same OSCC specimens, indicating that some cancer cells exhibit co-expression of these three marker proteins. Interestingly, the co-expression of all three proteins was more frequently observed in moderately or poorly differentiated OSCCs (Figs. 1b and 2c).

### Association between clinicopathological tumor features and the expression patterns of NANOG, mutant p53, and CD44

**Fig. 4:**
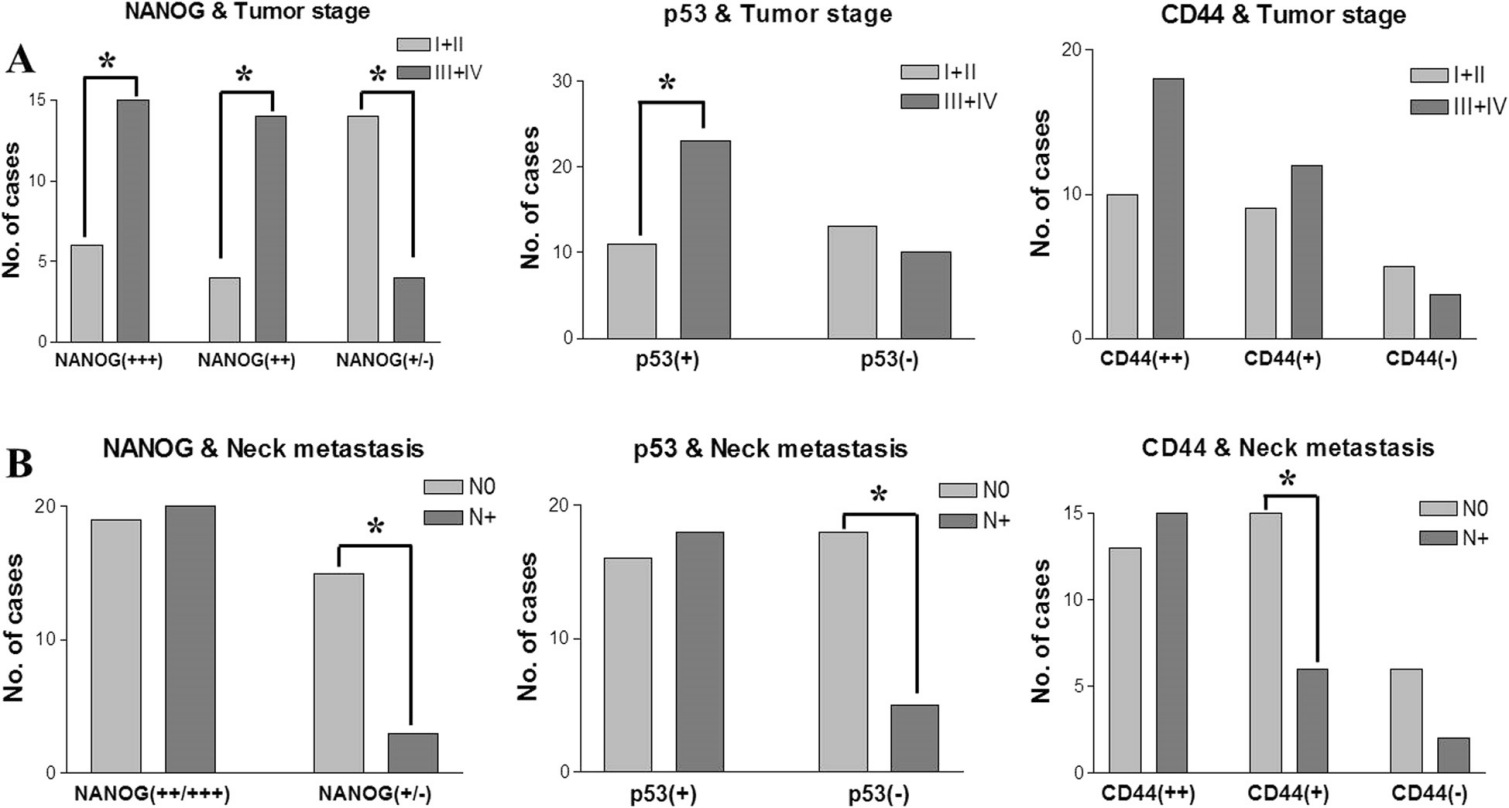
Correlation of tumor stage or neck node metastasis and the immunostaining patterns of NANOG, mutant p53, and CD44. **a** Strong or moderate expression of NANOG is directly associated with late-stage tumors, whereas weak or negative expression of NANOG is directly related to early-stage carcinoma (*p* < 0.05). Positive expression of mutant p53 is also significantly associated with late-stage tumors (*p* = 0.039). Higher expression of CD44 is frequently associated with late-stage tumors, but no statistical significance was observed (*p* > 0.05). **b** Tumors with weak or negative expression of NANOG (*p* = 0.004), negative expression of mutant p53 (*p* = 0.006), and weak expression of CD44 (*p* = 0.049) show a significantly higher frequency of negative neck node metastasis. Higher expression levels of the three proteins are not associated with neck node metastasis (*p* > 0.05). Data represent the number of cases with positive expression of each protein, and an asterisk (*) indicates a significant difference (*p* < 0.05). [Abbreviations: I + II, tumor stage I & II; III + IV, tumor stage III & IV; N0, negative neck node metastasis; N+, positive neck node metastasis]

Strong expression of NANOG was significantly correlated with histologically high-grade OSCCs (*p* < 0.001), whereas weak or negative expression of NANOG predominantly occurred in well-differentiated carcinomas (*p* = 0.018). Similarly, positive mutant p53 expression was significantly associated with high-grade carcinomas (*p* < 0.001) (Fig. 3b). High-grade tumors exhibited enhanced expression of CD44, but this finding was not statistically significant (Fig. 3b). In the assessment of the relationship between protein expression and tumor stage, strong and moderate expression of NANOG displayed direct associations with late-stage tumors (stage III and IV; *p* < 0.05), whereas weak expression of NANOG was directly associated with early-stage tumors (stage I and II; *p* = 0.018) (Fig. 4a). Positive mutant p53 expression was frequently observed in late-stage tumors (*p* = 0.049). Similarly, higher expression of CD44 was frequently observed in late-stage tumors, but the difference was not statistically significant (Fig. 4a). In the analysis of neck node metastasis, weak or negative expression of NANOG was significantly related to negative neck node metastasis in OSCCs (*p* = 0.005) (Fig. 4b). Similarly, negative expression of mutant p53 (*p* = 0.007) and weak expression of CD44 (*p* = 0.049) were significantly related to a higher frequency of negative neck node metastasis (Fig. 4b).

### Survival analysis

**Fig. 5:**
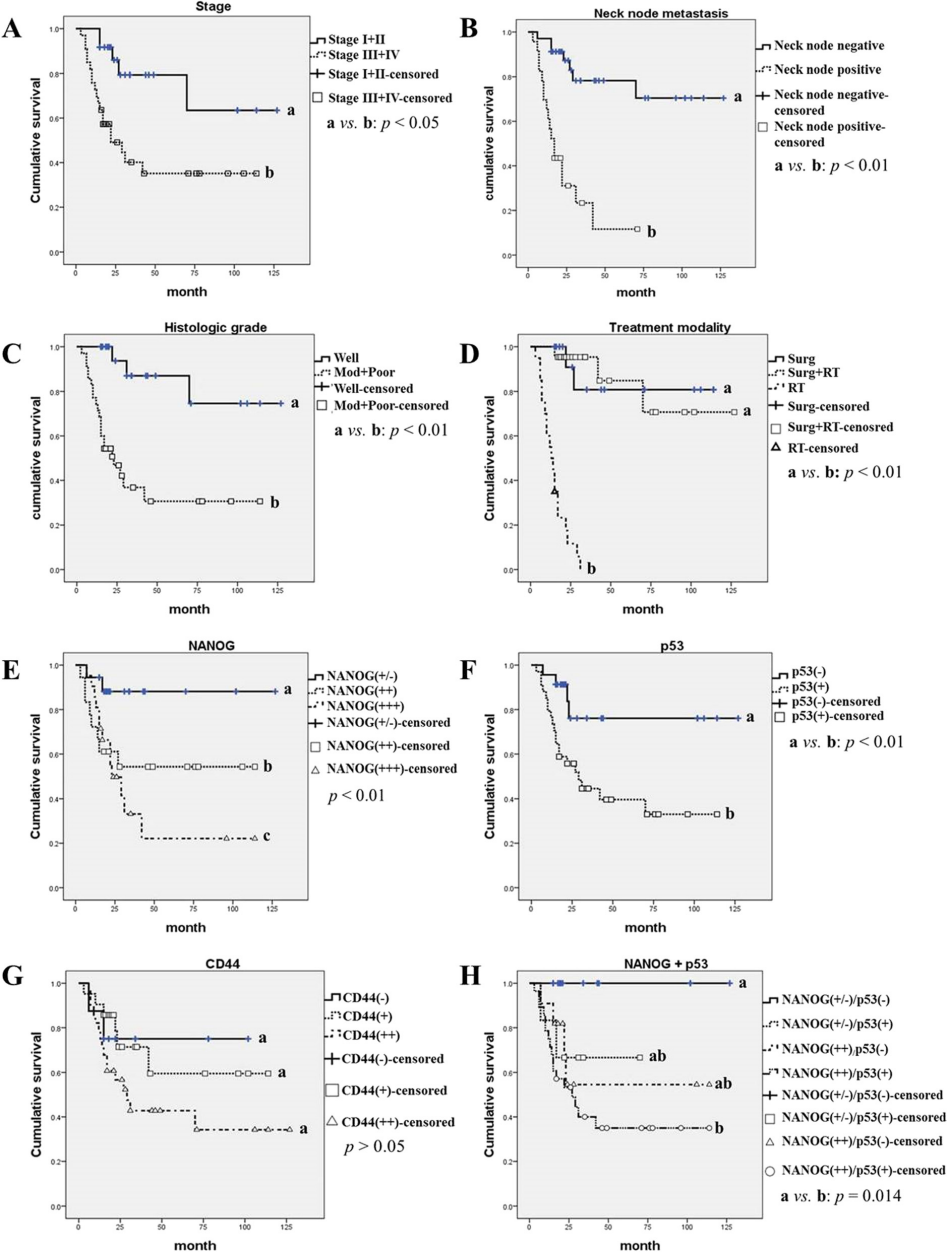
The overall survival rate of OSCC patients according to clinicopathological tumor features, treatment modality, and immunostaining patterns of NANOG, mutant p53, and CD44. **a** Patients with late-stage tumors (stage III & IV) show significantly poorer survival rates (*p* < 0.05). **b**–**d** A positive association between the overall survival rate of OSCC patients and histopathological tumor grade, neck node metastasis, and treatment modality is observed. The well-differentiated OSCC group, negative neck node group, and surgical treatment group show significantly better long-term survival rates than the corresponding opposite groups (*p* < 0.01). **e** Enhanced expression of NANOG is associated with a poor survival rate; in particular, patients with strong or moderate expression of NANOG show significantly lower survival rates than those with weak or negative NANOG expression (*p* < 0.01). **f** Patients with mutant p53(+)-expressing tumors show a lower survival rate than those with tumors negative for mutant p53(−) (*p* < 0.01). **g** Enhanced CD44 expression tends to correlate with poor survival rates, but no statistical significance was found (*p* > 0.05). **h** OSCCs with co-expression of enhanced NANOG and mutant p53 [NANOG(++)/p53(+)] correlate with a significantly lower overall survival rate than those with weak NANOG and p53 negativity [NANOG(+/−)/p53(−)] (*p* = 0.014). Notably, there were no deaths in the 12 cases of NANOG(+/−)/p53(−) during the follow-up period. Different letters denote statistically significant differences between groups (*p* < 0.05)

The analysis of overall patient survival rates in terms of clinicopathological tumor features revealed some significant results (Figs. 5a–d). Patients with late-stage OSCC (stage III and IV) had a statistically significant lower survival rate than those with early-stage tumors (Stage I and II) (*p* < 0.05, Fig. 5a). Patients with OSCC and positive neck node metastasis displayed a significantly poorer survival rate than those with negative neck node metastasis (*p* < 0.01, Fig. 5b). In addition, patients with histopathologically high-grade OSCC had a poorer prognosis than those with well-differentiated tumors (*p* < 0.01, Fig. 5c). In terms of treatment modality, the RT group exhibited a significantly poorer survival rate than the Surg and Surg + RT groups (*p* < 0.01, Fig. 5d).

Moreover, survival analysis in terms of the immunostaining patterns of NANOG, mutant p53, and CD44 revealed statistically significant differences (Figs. 5e–h). The expression intensity of NANOG was directly associated with the overall survival rate; patients with strong NANOG-expressing tumors [NANOG(+++)] had poorer survival rates than those with weak or negative NANOG-expressing tumors [NANOG(+/−)] (*p* < 0.01, Fig. 5e). Similarly, a positive association between mutant p53 expression and the overall survival rate was observed; specifically, patients with p53-positive OSCC had lower survival rates than those with p53-negative OSCC (*p* < 0.01, Fig. 5f). Enhanced CD44 expression was associated with poor survival rates, but this finding was not statistically significant (*p* > 0.05, Fig. 5g). Interestingly, patients with co-expression of enhanced NANOG (moderate or strong expression) and p53 positivity [NANOG(++)/p53(+)] had significantly shorter survival rates than patients with weak NANOG and negative p53 expression [NANOG(+/−)/p53(−)] (*p* = 0.014, Fig. 5h). Of significance, among the 28 patients with NANOG(++)/p53(+) tumors, 17 patients died, whereas there were no deaths in the 12 patients with NANOG(+/−)/p53(−) lesions during the follow-up period (Fig. 5h). Univariate Cox proportional hazards regression analysis revealed that neck node metastasis, histologically high-grade tumor, and late tumor stage were associated with significantly poorer prognosis. Moreover, enhanced expression of NANOG and mutant p53, especially co-detection of these two proteins, in the OSCC pretreatment biopsy specimens was directly associated with lower survival rates (Table 2). Similarly, the multivariate Cox proportional hazards regression model illustrated that neck node metastasis, histological grade, and immunohistochemical expression pattern of NANOG were directly related to the overall survival rate of patients with OSCC (Table 3).

**Table 2:** Univariate Cox proportional hazards regression analysis in relation to overall survival of the 57 OSCC patients

	Overall survival		
Variable	*P* value	Hazard ratio	95 % CI
Neck node	<0.001*	6.795	2.745–16.817
Histological grade	0.003*	12.271	2.336–64.465
Tumor stage	0.009*	3.718	1.385–9.980
NANOG	0.019*	4.494	1.275–15.839
Mutant p53	0.016*	3.747	1.279–10.977
CD44	0.263	2.315	0.532–10.097
NANOG + p53	0.028*	0.741	0.217–2.531

Abbreviation: *CI* confidence interval

*Statistically significant (*p* < 0.05)

**Table 3:** Multivariate Cox proportional hazards regression analysis in relation to overall survival of the 57 OSCC patients

	Overall survival		
Variable	*P* value	Hazard ratio	95 % Cl
Neck node	0.004*	6.549	1.800–23.822
Histological grade	0.012*	3.598	1.330–9.937
Tumor stage	0.254	3.546	0.403–31.225
NANOG	0.035*	4.319	3.351–27.476
Mutant p53	0.180	1.195	1. 713–5.754

Abbreviation: *CI* confidence interval

*Statistically significant (*p* < 0.05)

## Discussion

Since the emergence of the CSC hypothesis as a key theory to explain cancer progression, the expression patterns of several pluripotent stem cell markers and transcription factors have been studied in various cancer tissues or cell lines to determine their correlation with long-term prognosis. In the present study, the early transcription factor NANOG, the CSC marker CD44, and human mutant p53 were immunohistochemically investigated in the pretreatment biopsy specimens of 57 patients with OSCC to identify any relationship of their expression patterns with clinicopathological tumor features and patient prognosis. The results of this study demonstrated that the immunohistochemical expression patterns of NANOG and mutant p53 were directly associated with overall survival rates as well as clinicopathological features, including tumor stage, neck node metastasis, and histological grade. Patients with OSCC whose tumors had higher expression of NANOG and mutant p53 had less favorable clinicopathological features and lower survival rates. Enhanced expression of CD44 was also associated with poor prognosis and lower survival rates, but no statistical significance was observed. Recently, a common modality for predicting cancer prognosis has been the measurement of intra-tumor genetic heterogeneity [19, 20]. Heterogeneity denotes that tumors are composed of multiple clonal subpopulations of different characteristic cells, and thus, histological examination frequently reveals differences in cell morphology and properties, among cells in the same cancer specimen [20]. Enhanced intra-tumor heterogeneity was directly correlated with increased mortality; in particular, high heterogeneity and p53 mutation positivity were associated with higher mortality rates in a large cohort study of head and neck cancer [19]. In the present study, serial sections of tumor specimens frequently revealed the co-expression of NANOG, mutant p53, and CD44, which was associated with poorer prognosis. The co-detection of these different marker proteins in the same cancer cell or same tumor region may represent high tumor heterogeneity.

NANOG is an early transcription factor and pluripotent marker that maintains the self-renewal of embryonic and mesenchymal stem cells [8]. Recent studies revealed that NANOG is highly detected in poorly differentiated carcinomas and late-stage tumors [8–10]. Moreover, a positive relationship between enhanced NANOG expression and lymph node metastasis of carcinoma was reported [21]. Similarly, the present study illustrated that strong NANOG expression was positively correlated with clinically late-stage and histopathologically high-grade tumors. However, our findings on the relationship between neck node metastasis and NANOG expression slightly differed from those observed in previous studies. Weak or negative NANOG expression was associated with negative neck node metastasis, whereas higher NANOG expression was not associated with positive neck node metastasis in the present study. In IHC, NANOG is usually detected in the nuclei of pluripotent cells. However, in the present study, this transcription-related protein was sometimes observed in the cell cytoplasm, which is in agreement with previous reports in which NANOG was occasionally detected in the cytoplasm of highly primitive undifferentiated stem cells or tumor cells from patients with poor prognoses; these findings may be related to the pluripotency of stem cells and invasive or recurrent cancer cells [22, 23]. In this study, cytoplasmic NANOG expression was usually detected in high-grade OSCCs (Figs. 1 and 2). In addition, the overall survival rate was significantly lower in patients with strong NANOG expression than in those with weak or negative NANOG expression. These results suggest that OSCCs with high NANOG expression, including cytoplasmic expression, may possess aggressive characteristics, which in turn indicate poor prognosis.

The wild-type p53 protein is a DNA-binding transcription factor that acts as a tumor suppressor by allowing the cell time to repair and recover from DNA damage or by inducing cell apoptosis in cases of serious damage [12, 24]. A review of a large cohort study of OSCC revealed conflicting evidence for p53 as a tumor prognostic factor; some studies uncovered that positive p53 staining was associated with poor prognosis, whereas others reported no such association [1]. These contradictory results may be related to the frequency of mutation of the p53 gene in cancer tissues, particularly carcinomas. Mutant p53 causes the inactivation and dysfunction of wild-type p53 and plays a pivotal role in the development and progression of carcinomas [13, 25]. Co-detection of p53 and mouse double minute 2 (MDM2) by IHC might be helpful for discriminating the functional type of p53; high levels of p53 without increased MDM2 expression may indicate the inactivating type of p53, usually mutant p53 [17]. Interestingly, mutant p53 is easily detected via immunohistochemical analysis [14]. Although wild-type p53 can also be detected in cancerous tissues using IHC, wild-type p53 (normally a tumor suppressor gene) may be not related with poor prognosis of cancer [26, 27]. In the present study, a human anti-mutant p53 antibody (Abcam™, ab32049), which does not recognize human wild-type p53 protein but does react with a synthetic peptide corresponding to human mutant p53 aa 1-100 (N terminal), was used to detect the mutated type of p53 in the pretreatment biopsy specimens of patients with OSCC. A positive p53 mutation signal was detected in 34 of 57 OSCC specimens (59.6 %). This result supports the finding from a previous study, which reported a p53 mutation rate of 40–60 % in carcinoma tissues [1, 17]. In addition, we observed a positive correlation between high NANOG expression and mutant p53 positivity: OSCCs with moderate or strong expression of NANOG had a higher frequency of p53 mutation (Fig. 3a). In addition, OSCCs that expressed mutant p53 were more frequently high-grade tumors (Fig. 3b), whereas tumors that were negative for mutant p53 were associated with fewer neck node metastases (Fig. 4b). Moreover, patients with p53 mutations had a significantly poorer survival rate than those without p53 mutations (Fig. 5f). Interestingly, the co-detection of an enhanced NANOG signal and mutant p53 [NANOG(++)/p53(+)] was correlated with significantly lower patient survival versus tumors with weak NANOG expression and p53 negativity [NANOG(+/−)/p53(−)] (Fig. 5h).

CD44 is a cell surface glycoprotein that acts as a receptor for hyaluronic acid and as an adhesion molecule [7, 28]. This cell surface protein plays a role in tumor cell invasion, metastasis, and angiogenesis by interacting with certain matrix metalloproteinases [29]. CD44 was first described as a CSC marker in breast cancer and HNSCC [4, 5], and it has since been used as a CSC marker and prognostic factor for SCC [11, 30]. CD44 has exhibited positive correlations with tumor recurrence, high-grade SCCs, and poor prognosis [30]. However, its limited usefulness as a CSC marker or prognostic factor in OSCC was also reported [31, 32]. In the present study, OSCCs that expressed high levels of NANOG and mutant p53 also displayed significantly elevated expression of CD44 (Fig. 3a). In addition, high CD44 expression was associated with clinically late-stage and histologically high-grade tumors, although no statistical significance was found in this analysis. Only the group with weak CD44 expression displayed a statistically higher frequency of negative neck node metastasis (Fig. 4b). However, in this study, the CD44 expression pattern was not statistically correlated with overall survival rates, but high expression of CD44 tended to be associated with poor prognosis (Fig. 5g). These results indicate that CD44 expression could have limited use as a clinicopathological and prognostic marker for OSCC, as suggested in previous reports [28, 32].

In this study, overall survival rates were analyzed in terms of the clinicopathological features of OSCCs as well as the immunohistochemical expression patterns of the three marker proteins. In the Kaplan-Meier analysis with the log-rank test, patients with late-stage tumors, positive neck node metastases, and high histopathological grade tumors had significantly lower survival rates. Similar results were obtained by univariate and multivariate Cox proportional hazards regression analysis for overall survival of the 57 patients with OSCC. Neck node metastasis, histological grade, tumor stage, and the expression intensity of NANOG and mutant p53 were directly associated with the overall survival rate of the patients; however, the relationship between survival and the expression pattern of mutant p53 was not statistically significant in multivariate Cox analysis (Tables 2–3). In the three groups as divided treatment modality, the RT group displayed a much poorer prognosis than the Surg or Surg + RT group (Fig. 5d). This result is likely attributable to the characteristics of the patients in the RT group, as palliative radiotherapy is selected as a treatment option in cases of tumor inoperability or patient refusal to undergo surgery.

## Conclusions

Although this study has a limited number of cases and used only IHC in paraffin-embedded tissue specimens, the results suggest that co-detection of mutant p53 and high NANOG levels in the pretreatment biopsy specimens of patients with OSCC is directly associated with unfavorable clinicopathological tumor features and poor survival rates. The enhanced expression of CD44 also displayed a limited correlation with clinically late-stage and histopathologically high-grade OSCCs. Taken together, the immunohistochemical expression patterns of NANOG, mutant p53, and CD44 in pretreatment biopsy specimens could be used as predictive markers for prognosis and tumor aggressiveness in patients with OSCC. Particularly, analysis for co-expression of NANOG and mutant p53 could be used as a tool for prognosis and for selecting individual treatment modalities.
